# Supplementary material for: Restoring cellular magnesium balance through Cyclin M4 protects against acetaminophen-induced liver damage
Source: Nat Commun. 2022 Nov 25;13:6816. doi: 10.1038/s41467-022-34262-0 (PMC9700862; doi:10.1038/s41467-022-34262-0)
Supplement: Supplementary file 1 — Supplementary Information [file 41467_2022_34262_MOESM1_ESM.pdf]

SUPPLEMENTAL FIGURE 1

a

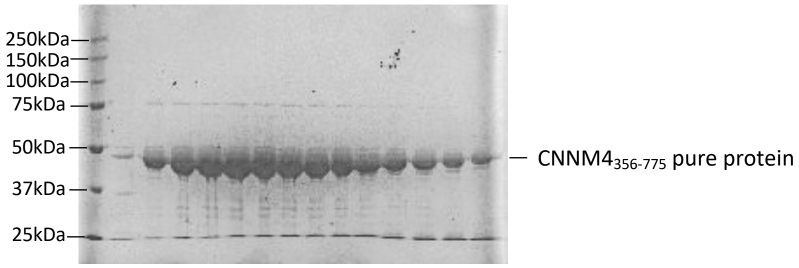

b

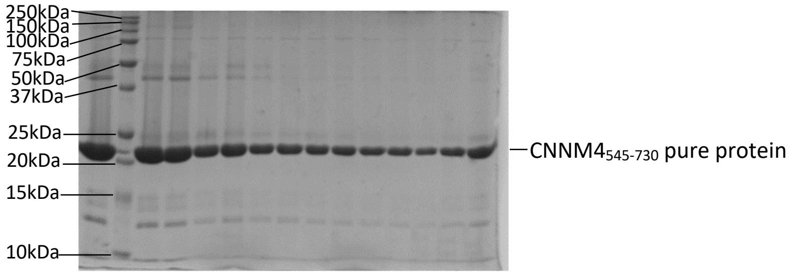

Supplemental Figure 1. Purified protein of $CNNM4_{BATEMAN\text{-}cNMP\text{-}Ctail}$ and $CNNM4_{cNMP}$.

A. The purity of $CNNM4_{BATEMAN\text{-}cNMP\text{-}Ctail}$ was confirmed in a SDS-polyacrylamide gel from a gel filtration chromatography. The theoretical molecular weight of the protein was 47.86 kDa. **B.** The purity of $CNNM4_{cNMP}$ was confirm in a SDS-polyacrylamide gel from a gel filtration chromatography. The theoretical molecular weight of the protein was 20.94 kDa. These experiments were repeated independently three times.

a

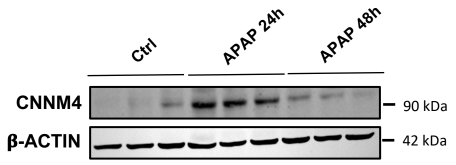
Ctrl
APAP 24h
APAP 48h
CNNM4
β-ACTIN
90 kDa
42 kDa

b

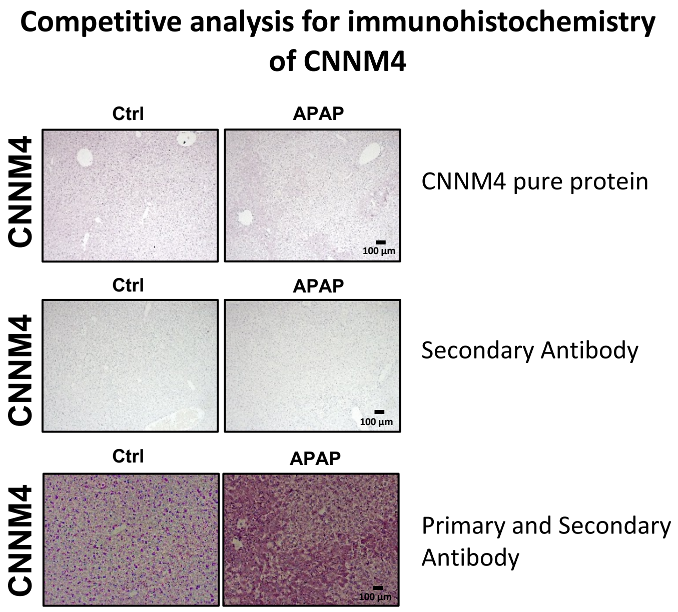
Competitive analysis for immunohistochemistry of CNNM4
Ctrl
APAP
CNNM4
100 µm
CNNM4 pure protein
Secondary Antibody
Primary and Secondary Antibody

Supplemental Figure 2. Cyclin M4 (CNNM4) is overexpressed pre-clinical animal models caused by APAP.

A. Western blot analysis of CNNM4 in animals treated with a single dose of APAP 360mg/kg for 24h (n=4) and 48h (n=4) compared to a healthy group (n=4). β-ACTIN was used as a loading control. **B.** Competitive analysis in liver tissue of mice (n=3) for immunohistochemistry of CNNM4 in three different conditions: IHC with 0.3 μmol/mL of CNNM4 pure protein, with only secondary antibody and with primary and secondary antibody. These experiments were repeated independently three times. Source data are provided as a Source Data file.

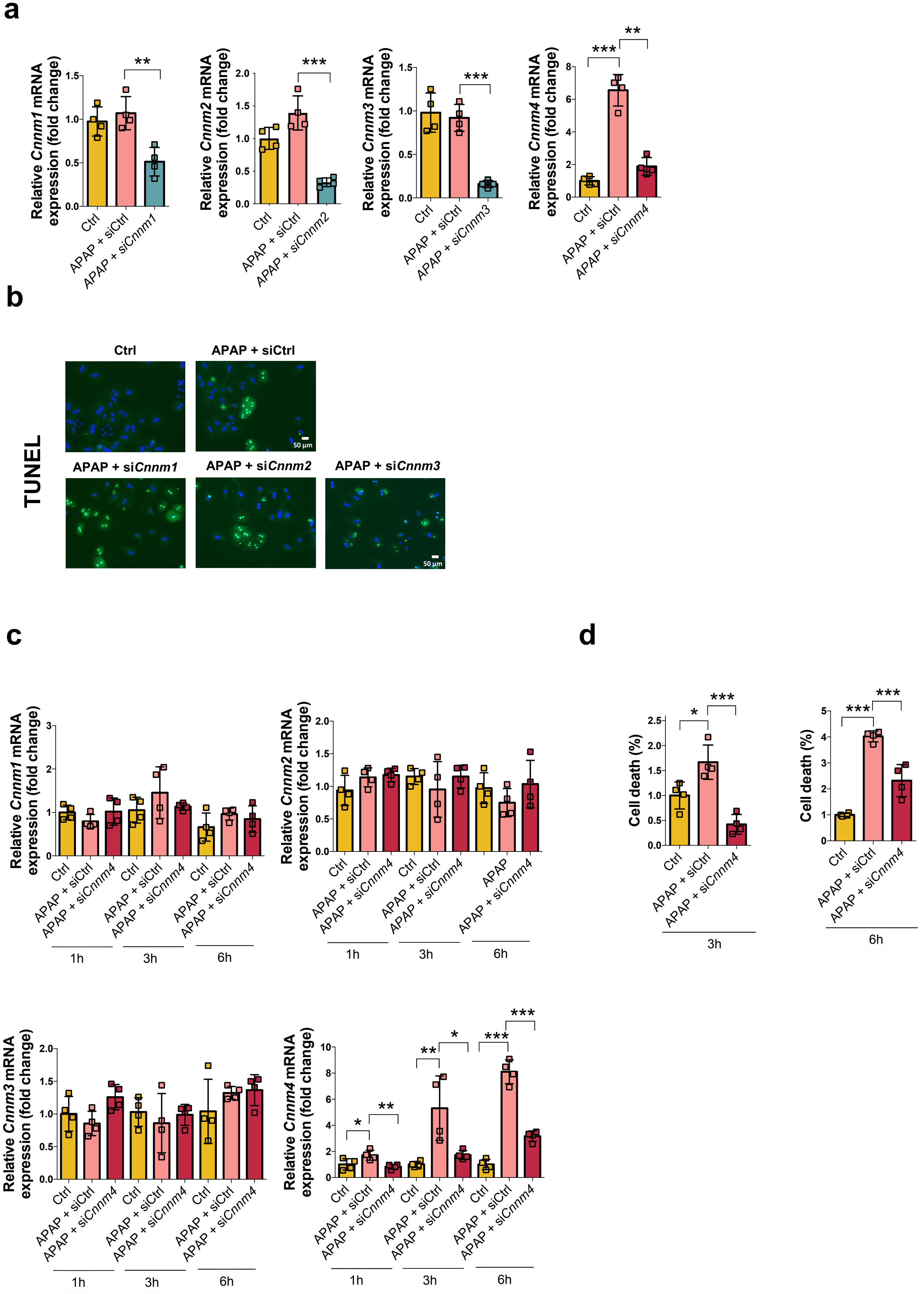
a
Relative Cnnm1 mRNA expression (fold change)
Ctrl
APAP + siCtrl
APAP + siCnnm1
Relative Cnnm2 mRNA expression (fold change)
APAP + siCnnm2
Relative Cnnm3 mRNA expression (fold change)
APAP + siCnnm3
Relative Cnnm4 mRNA expression (fold change)
APAP + siCnnm4
b
TUNEL
Ctrl
APAP + siCtrl
APAP + siCnnm1
APAP + siCnnm2
APAP + siCnnm3
50 µm
c
1h
3h
6h
APAP
d
Cell death (%)
3h
6h
*
**
***

Supplemental Figure 3. Silencing *Cnnm4* protects against cell death caused by APAP toxicity in primary hepatocytes.

A. mRNA levels of *Cnnm1*, *Cnnm2*, *Cnnm3* and *Cnnm4* in WT hepatocytes under APAP overdose for 3h and treated with a siRNA *Cnnm1*, *Cnnm2*, *Cnnm3* and *Cnnm4* or an unrelated control (siCtrl) compared to a control group (Ctrl). Values are represented as mean ± SEM. ***P* < 0.01; ****P* < 0.001 (Cnnm1 *P*=0.0044 APAP+si*Cnnm1* vs APAP) (Cnnm2 *P*=0.00022 APAP+si*Cnnm2* vs APAP) (Cnnm3 *P*=7.93E-05 APAP+si*Cnnm3* vs APAP) (Cnnm4 *P*=3.13E-05 APAP vs Ctrl; *P*=0.00015 APAP+si*Cnnm4* vs APAP) (Student's test, two-sided). **B.** Cell death was evaluated using TUNEL in WT hepatocytes under APAP overdose for 3h and treated with a siRNA *Cnnm1*, *Cnnm2*, *Cnnm3* and *Cnnm4* or an unrelated control (siCtrl) compared to a control group (Ctrl). Scale bar correspond to 50 μm **C.** mRNA levels of *Cnnm1*, *Cnnm2*, *Cnnm3* and *Cnnm4* for 1h, 3h and 6h under APAP overdose and treated with a siRNA *Cnnm4* or an unrelated control (siCtrl) compared to a control group (Ctrl). Values are represented as mean. Values are represented as mean ± SEM. **P* < 0.05; ***P* < 0.01; ****P* < 0.001 (1h *P*=0.05 APAP vs Ctrl; *P*=0.006 APAP+si*Cnnm4* vs APAP) (3h *P*=0.01 APAP vs Ctrl; *P*=0.028 APAP+si*Cnnm4* vs APAP) (6h *P*=7.38E-06 APAP vs Ctrl; *P*=6.22E-05 APAP+si*Cnnm4* vs APAP) (Student's test, two-sided). **D.** Cell death was evaluated using Trypan Blue in WT hepatocytes under APAP overdose for 3h and 6h and treated with a siRNA *Cnnm4* or an unrelated control (siCtrl) compared to a control group (Ctrl). Quadrupled were used for experimental conditions. Values are represented as mean ± SEM. **P* < 0.05, ***P* < 0.01, ****P* < 0.001 (3h *P*=0.022 APAP vs Ctrl; *P*=0.00076 APAP+si*Cnnm4* vs APAP) (6h *P*=1.66E-07 APAP vs Ctrl; *P*=0.001 APAP+si*Cnnm4* vs APAP) (Student's test, two-sided). Source data are provided as a Source Data file.

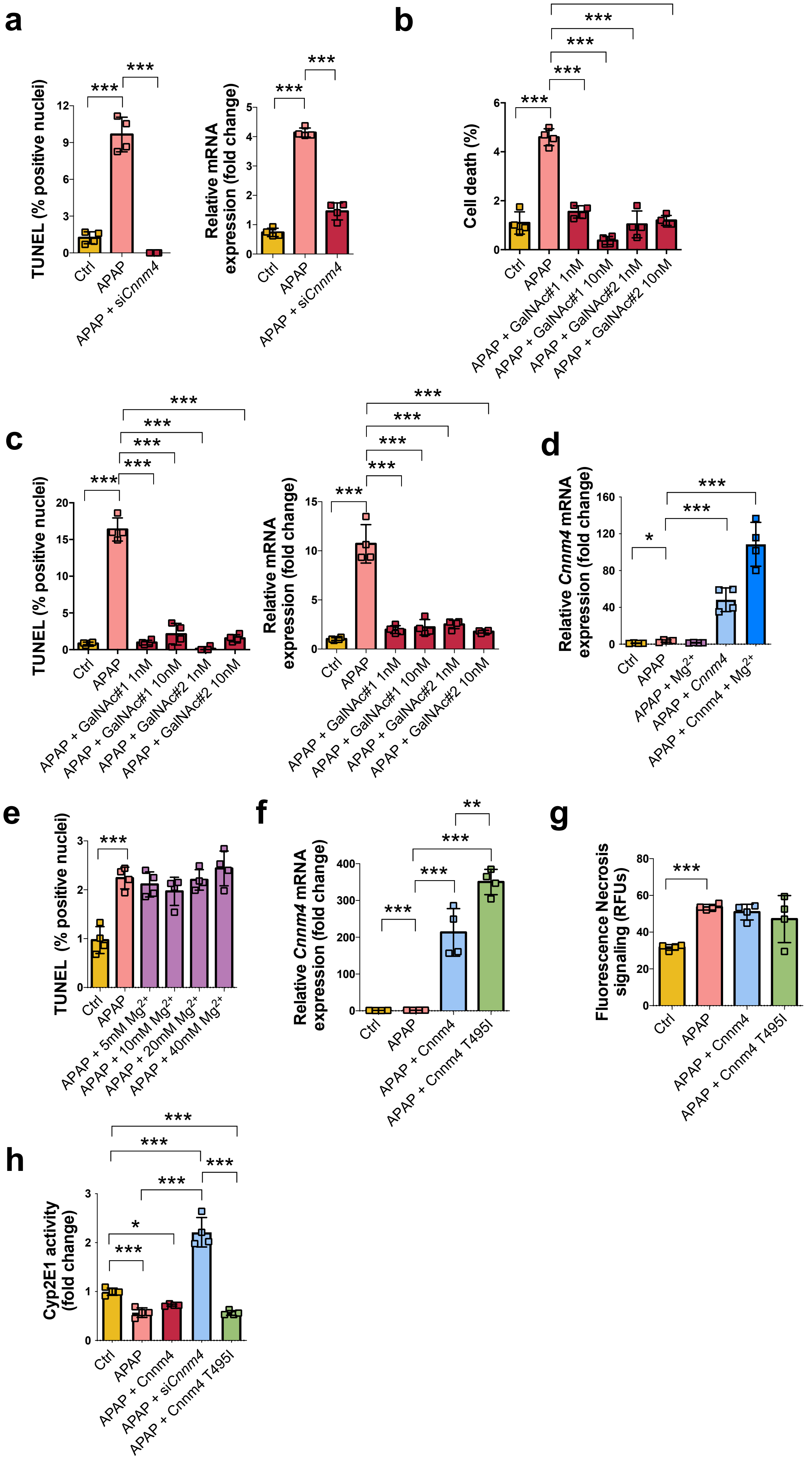
a
TUNEL (% positive nuclei)
Relative mRNA expression (fold change)
Ctrl
APAP
APAP + siCnnm4
b
Cell death (%)
APAP + GalNAc#1 1nM
APAP + GalNAc#1 10nM
APAP + GalNAc#2 1nM
APAP + GalNAc#2 10nM
c
d
Relative Cnnm4 mRNA expression (fold change)
APAP + Mg2+
APAP + Cnnm4
APAP + Cnnm4 + Mg2+
e
APAP + 5mM Mg2+
APAP + 10mM Mg2+
APAP + 20mM Mg2+
APAP + 40mM Mg2+
f
APAP + Cnnm4 T495I
g
Fluorescence Necrosis signaling (RFUs)
h
Cyp2E1 activity (fold change)

Supplemental Figure 4. Mg^{2+} supplementation failed to solve APAP-mediated toxicity

A. Cell death was evaluated using TUNEL in THLE2 cell line under APAP overdose for 6h and treated with a siRNA *Cnnm4* or an unrelated control (siCtrl) compared to a control group (Ctrl). Values are represented as mean ± SEM. ****P* < 0.001 (TUNEL *P*=2.87E-05 APAP vs Ctrl; *P*=9.37E-06 APAP+si*Cnnm4* vs APAP) (mRNA *P*=5.72E-08 APAP vs Ctrl; *P*=3.28E-06 APAP+si*Cnnm4* vs APAP) (Student's test, two-sided). **B.** Cell death was evaluated using Trypan Blue in WT hepatocytes under APAP overdose for 6h and treated with two GalNAc siRNA *Cnnm4* (GalNAc#1 and GalNAc#2) molecules at different concentrations 1nM and 10nM compared to a control group (Ctrl). Values are represented as mean ± SEM. ****P* < 0.001 (*P*=1.79E-05 APAP vs Ctrl; *P*=7.41E-06 APAP+GalNAc#1 1nM vs APAP; *P*=5.63E-07 APAP+GalNAc#1 10nM vs APAP; *P*=3.35E-05 APAP+GalNAc#2 1nM vs APAP; *P*=2.66E-06 APAP+GalNAc#2 10nM vs APAP) (Student's test, two-sided). **C.** Cell death was evaluated using TUNEL in WT hepatocytes under APAP overdose for 6h and with two GalNAc siRNA *Cnnm4* (GalNAc#1 and GalNAc#2) molecules at different concentrations 1nM and 10nM compared to a control group (Ctrl). Values are represented as mean ± SEM. ****P* < 0.001 (TUNEL *P*=1.15E-06 APAP vs Ctrl; *P*=1.31E-06 APAP+GalNAc#1 1nM vs APAP; *P*=1.16E-05 APAP+GalNAc#1 10nM vs APAP; *P*=9.28E-07 APAP+GalNAc#2 1nM vs APAP; *P*=1.92E-06 APAP+GalNAc#2 10nM vs APAP) (mRNA *P*=6.14E-05 APAP vs Ctrl; *P*=0.00012 APAP+GalNAc#1 1nM vs APAP; *P*=0.00019 APAP+GalNAc#1 10nM vs APAP; *P*=0.00018 APAP+GalNAc#2 1nM vs APAP; *P*=9.71E-05 APAP+GalNAc#2 10nM vs APAP) (Student's test, two-sided). **D.** mRNA *Cnnm4* expression levels in WT hepatocytes under APAP overdose for 3h with and without 5mM of Mg^{2+}, overexpressing CNNM4 and overexpressing CNNM4 with Mg^{2+} compared to a control group (Ctrl). Values are represented as mean ± SEM. ***P* < 0.01; ****P* < 0.001 (*P*=0.0084 APAP vs Ctrl; *P*=0.00043 APAP+Cnnm4 vs APAP; *P*=0.00012 APAP+Cnnm4+Mg^{2+} vs APAP) (Student's test, two-sided). **E.** Cell death was evaluated using TUNEL in WT primary hepatocytes under APAP overdose for 3h and supplemented with 5mM, 10mM, 20mM and 40mM Mg^{2+}. Values are represented as mean ± SEM. ****P* < 0.001 (*P*=0.00037 APAP vs Ctrl) (Student's test, two-sided). **F.** mRNA *Cnnm4* expression levels in WT primary hepatocytes under APAP overdose and overexpression Cnnm4 and Cnnm4 T495I. Values are represented as mean ± SEM. ****P* < 0.001 (*P*=0.0007 APAP vs Ctrl; *P*=0.00061 APAP+Cnnm4 vs APAP; *P*=9.24E-07 APAP+Cnnm4 T495I vs APAP; *P*=0.0096) (Student's test, two-sided). **G.** Cell death was evaluated using Annexin V Apoptosis and necrosis assay in WT hepatocytes under APAP overdose for overexpressing Cnnm4 and overexpressing the mutant Cnnm4 T495I and compared to a control group (Ctrl). Values are represented as mean ± SEM. ****P* < 0.001 (*P*=9.49E-07 APAP vs Ctrl) (Student's test, two-sided). **H.** Cyp2e1 activity was evaluated in WT primary hepatocytes under APAP overdose for 3h and treated with si*Cnnm4* RNA, Cnnm4 and Cnnm4 T495I overexpression. Quadrupled were used for experimental conditions. Values are represented as mean ± SEM. ***P* < 0.01; ****P* < 0.001 (*P*=0.00043 APAP vs Ctrl; *P*=0.02 APAP+Cnnm4 vs APAP; *P*=4.78E-05 APAP+si*Cnnm4* vs APAP) (Student's test, two-sided). Source data are provided as a Source Data file.

a

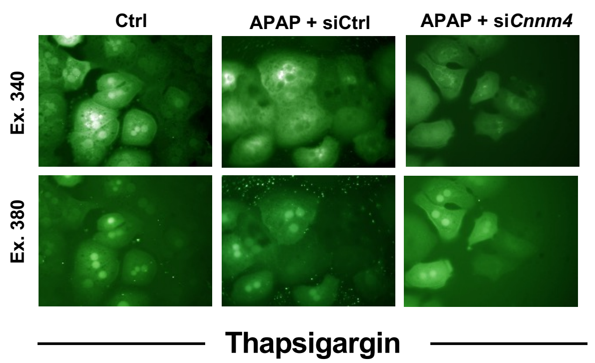
Ctrl
APAP + siCtrl
APAP + si*Cnnm4*
Ex. 340
Ex. 380
Thapsigargin
Ctrl
APAP + siCtrl
APAP + si*Cnnm4*
Ex. 340
Ex. 380
ATP

b

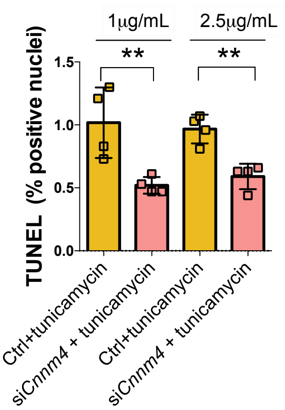
1µg/mL
2.5µg/mL
**
**
TUNEL (% positive nuclei)
1.5
1.0
0.5
0.0
Ctrl+tunicamycin
si*Cnnm4* + tunicamycin
Ctrl+tunicamycin
si*Cnnm4* + tunicamycin

Supplemental Figure 5. Silencing *Cnnm4* in hepatocytes reduces endoplasmic reticulum stress

A. Calcium (Ca^{2+}) release capacity by ER with thapsigargin and ATP upon time under APAP overdose for 3h treated with a siRNA *Cnnm4* or an unrelated control (siCtrl) compared to a healthy group (Ctrl). **B.** Cell death was evaluated using TUNEL assay in WT primary hepatocytes treated with two different doses of tunicamycin (1μg/mL and 2.5μg/mL) with si*Cnnm4* (n=4) or an unrelated control (n=4). Values are represented as mean ± SEM. ***P* < 0.01 (*P*=0.013 si*Cnnm4*+ 1μg/mL tunicamycin vs Ctrl 1μg/mL tunicamycin; *P*=0.0026 si*Cnnm4*+2.5μg/mL tunicamycin vs Ctrl) (Student's test, two-sided). Source data are provided as a Source Data file.

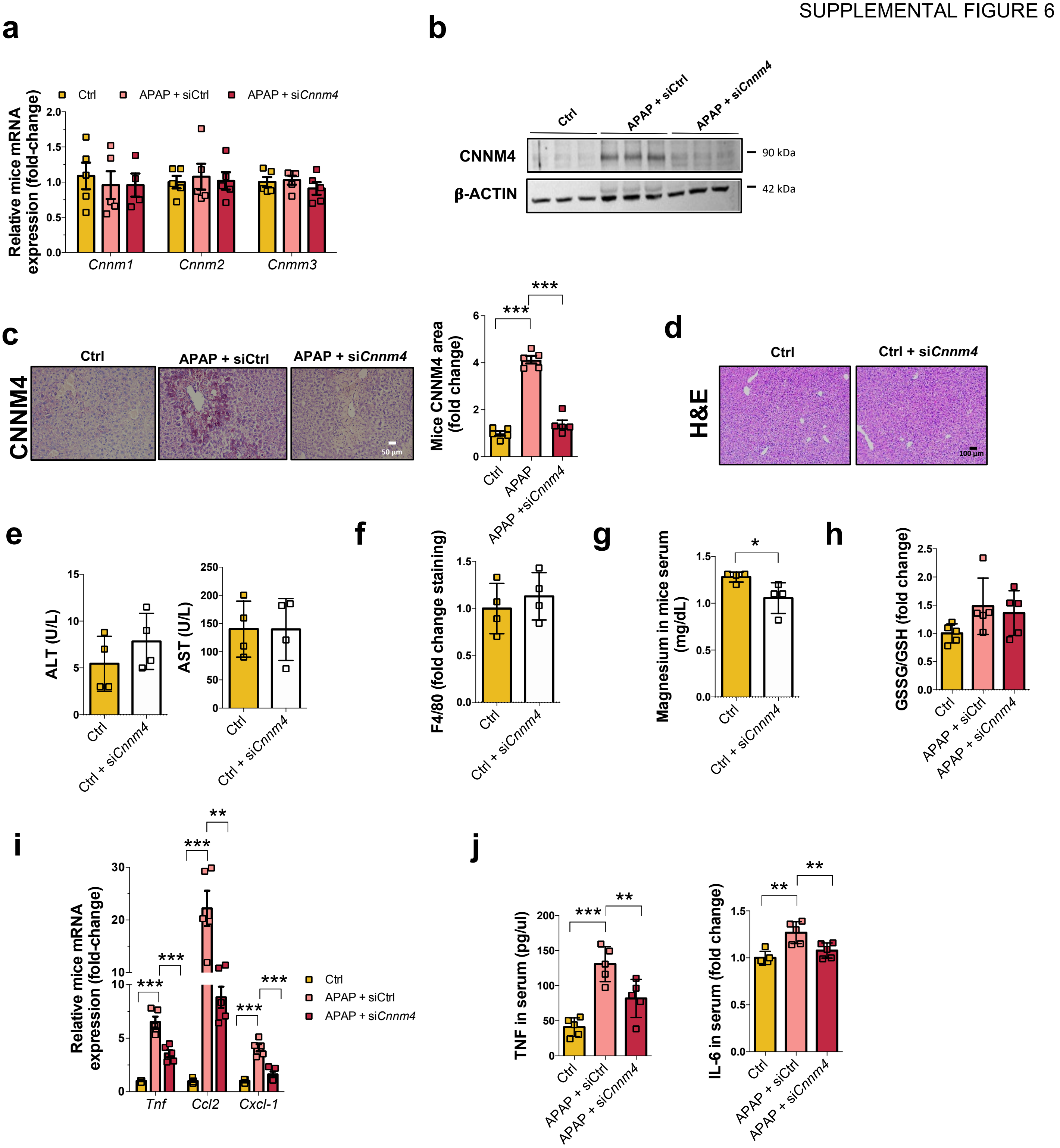
a
Ctrl
APAP + siCtrl
APAP + siCnnm4
Relative mice mRNA expression (fold-change)
Cnnm1
Cnnm2
Cnmm3
b
Ctrl
APAP + siCtrl
APAP + siCnnm4
CNNM4
β-ACTIN
90 kDa
42 kDa
c
CNNM4
Ctrl
APAP + siCtrl
APAP + siCnnm4
50 µm
Mice CNNM4 area (fold change)
APAP
APAP +siCnnm4
d
H&E
Ctrl + siCnnm4
100 µm
e
ALT (U/L)
AST (U/L)
f
F4/80 (fold change staining)
g
Magnesium in mice serum (mg/dL)
h
GSSG/GSH (fold change)
i
Relative mice mRNA expression (fold-change)
Tnf
Ccl2
Cxcl-1
j
TNF in serum (pg/ul)
IL-6 in serum (fold change)

Supplemental Figure 6. Silencing *Cnnm4* prevents APAP hepatotoxicity in *in vivo* models.

A-K. WT mice were treated with APAP 360 mg/kg by intraperitoneal injection 48 h and in the last 24h siRNA *Cnnm4* (n=5) or an unrelated control (siCtrl) (n=5) were injected via tail vein and compared to control group (n=5). **A.** mRNA levels of *Cnnm1, Cnnm2* and *Cnnm3*. **B.** Western Blot analysis of CNNM4, β-ACTIN was used as a loading control. **C.** Liver immunohistochemical staining and respective quantification of CNNM4 was determined in the experimental groups of APAP 360mg/kg for 48h (n=5) and silenced si*Cnnm4* or unrelated control animal models compared to a healthy group (n=5). Scale bar correspond to 50 µm. Values are represented as mean ± SEM. ****P* < 0.001 (*P*=1.87E-07 APAP vs Ctrl; *P*=2.92E-06 APAP+si*Cnnm4*) (Student's test, two-sided). **D-G**.WT mice were treated with siRNA *Cnnm4* (n=4) via tail vein injection and compared to control group (n=4). Mice were sacrificed 24h after. **D.** Liver necrosis was assessed by H&E staining. Scale bar correspond to 100 µm. **E.** Transaminases ALT and AST levels were determined in mice serum. **F.** Inflammation assessed by F4/80 staining in liver. **G.** Magnesium levels were determined in mice serum. Values are represented as mean ± SEM. **P* < 0.05 (*P*=0.046 Ctrl+si*Cnnm4* vs Ctrl) (Student's test, two-sided). **H.** GSSG/GSH levels measured by HPLC-MS. **I.** mRNA levels of *Tnf, Ccl2 and Cxcl-1*. Values are represented as mean ± SEM. **P* < 0.05; ***P* < 0.01; ****P* < 0.001 (*Tnf P*=4.99E-06 APAP vs Ctrl; *P*= 0.0014 APAP+si*Cnnm4* vs APAP) (*Ccl2 P*=0.00022 APAP vs Ctrl; *P*=0.005 APAP+si*Cnnm4* vs APAP) (*Cxcl-1 P*=1.64E-05 APAP vs Ctrl; *P*=0.00036 APAP+si*Cnnm4* vs APAP) (Student's test, two-sided). **J.** TNF and IL-6 levels were measured using ELISA assay in mice serum after 48h of APAP overdose. Values are represented as mean ± SEM. **P* < 0.05, ***P* < 0.01, ***P* < 0.001 (*TNF P*=0.0001 APAP vs Ctrl; *P*=0.01 APAP+si*Cnnm4* vs APAP) (IL6 *P*=0.0024 APAP vs Ctrl; *P*=0.017 APAP+si*Cnnm4* vs APAP) (Student's test, two-sided). Source data are provided as a Source Data file.

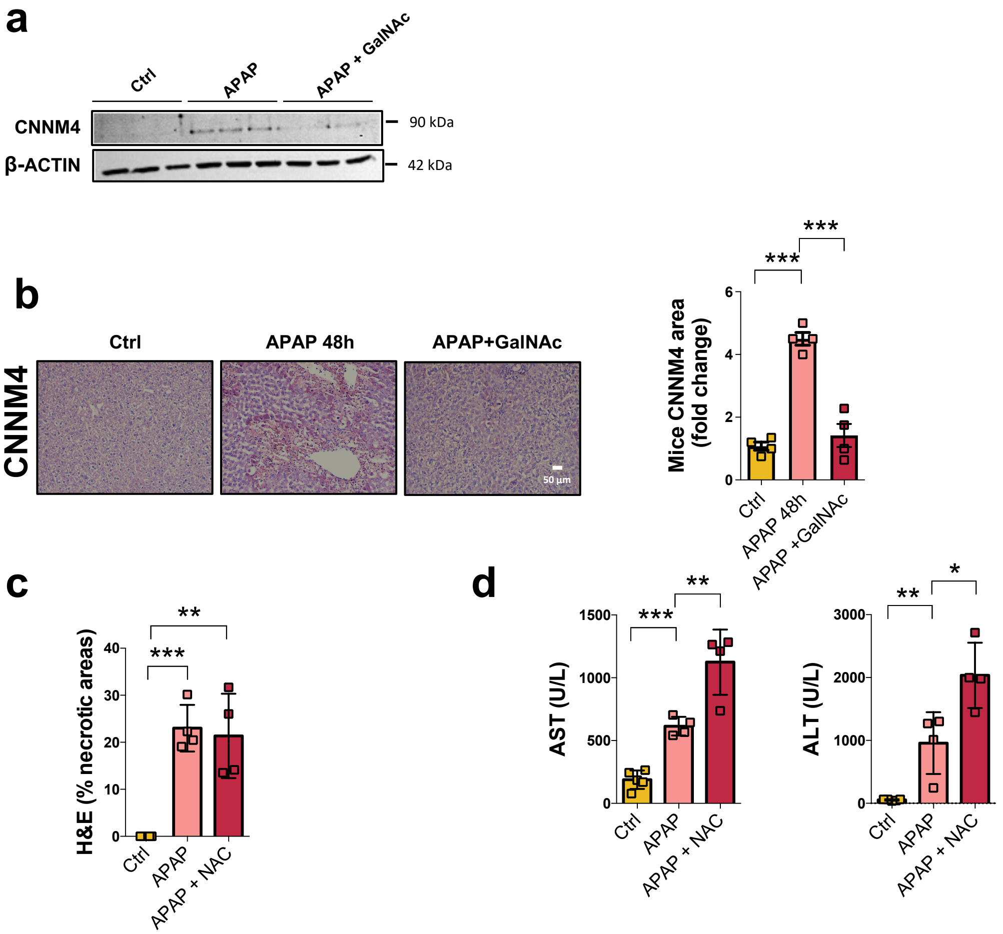

a
Ctrl
APAP
APAP + GalNAc
CNNM4
β-ACTIN
90 kDa
42 kDa
b
Ctrl
APAP 48h
APAP+GalNAc
CNNM4
50 µm
Mice CNNM4 area (fold change)
***
***
Ctrl
APAP 48h
APAP +GalNAc
c
H&E (% necrotic areas)
***
**
Ctrl
APAP
APAP + NAC
d
AST (U/L)
***
**
Ctrl
APAP
APAP + NAC
ALT (U/L)
**
*
Ctrl
APAP
APAP + NAC

Supplemental Figure 7. NAC is not a therapeutical option after 24 hours of APAP overdose

A-B**.** WT mice were treated with APAP 360 mg/kg by intraperitoneal injection 48 h and in the last 24h 3mg/kg GalNAc *Cnnm4* siRNA (n=4) or an unrelated control (n=4) were injected subcutaneously. Mice were sacrificed 48h after APAP overdose. **A.** Western Blot analysis of CNNM4, β-ACTIN was used as a loading control. **B.** Liver immunohistochemical staining and respective quantification of CNNM4 was determined in the experimental groups of APAP 360mg/kg for 48h (n=4) and silenced with GalNAc si*Cnnm4* or unrelated control (n=4) animal models compared to a healthy group (n=4). Scale bar correspond to 50 µm. Values are represented as mean ± SEM. ***P < 0.001 (P=7.76E-06 APAP vs Ctrl; P=0.00033 APAP+si*Cnnm4* vs APAP) (Student's test, two-sided). **C-D**. WT mice were treated with APAP 360 mg/kg by intraperitoneal injection (n=4) and in the last 24h mice received 1200mg/kg of NAC (n=4) by intraperitoneal injection and compared to control group. **C.** Liver necrosis was assessed by H&E staining. Values are represented as mean ± SEM. **P < 0.01; ***P < 0.001 (P=8.84E-05 APAP vs Ctrl; P=0.0031 APAP+NAC vs Ctrl) (Student's test, two-sided). **D.** Transaminases ALT and AST levels were determined in mice serum. Values are represented as mean ± SEM. *P< 0.05, **P<0.01, **P<0.001 (AST P=9.54E-05 APAP vs Ctrl; P= 0.0092 APAP+si*Cnnm4* vs APAP) (ALT P=0.01 APAP vs Ctrl; P= 0.024 APAP+si*Cnnm4* vs APAP) (Student's test, two-sided). Source data are provided as a Source Data file.

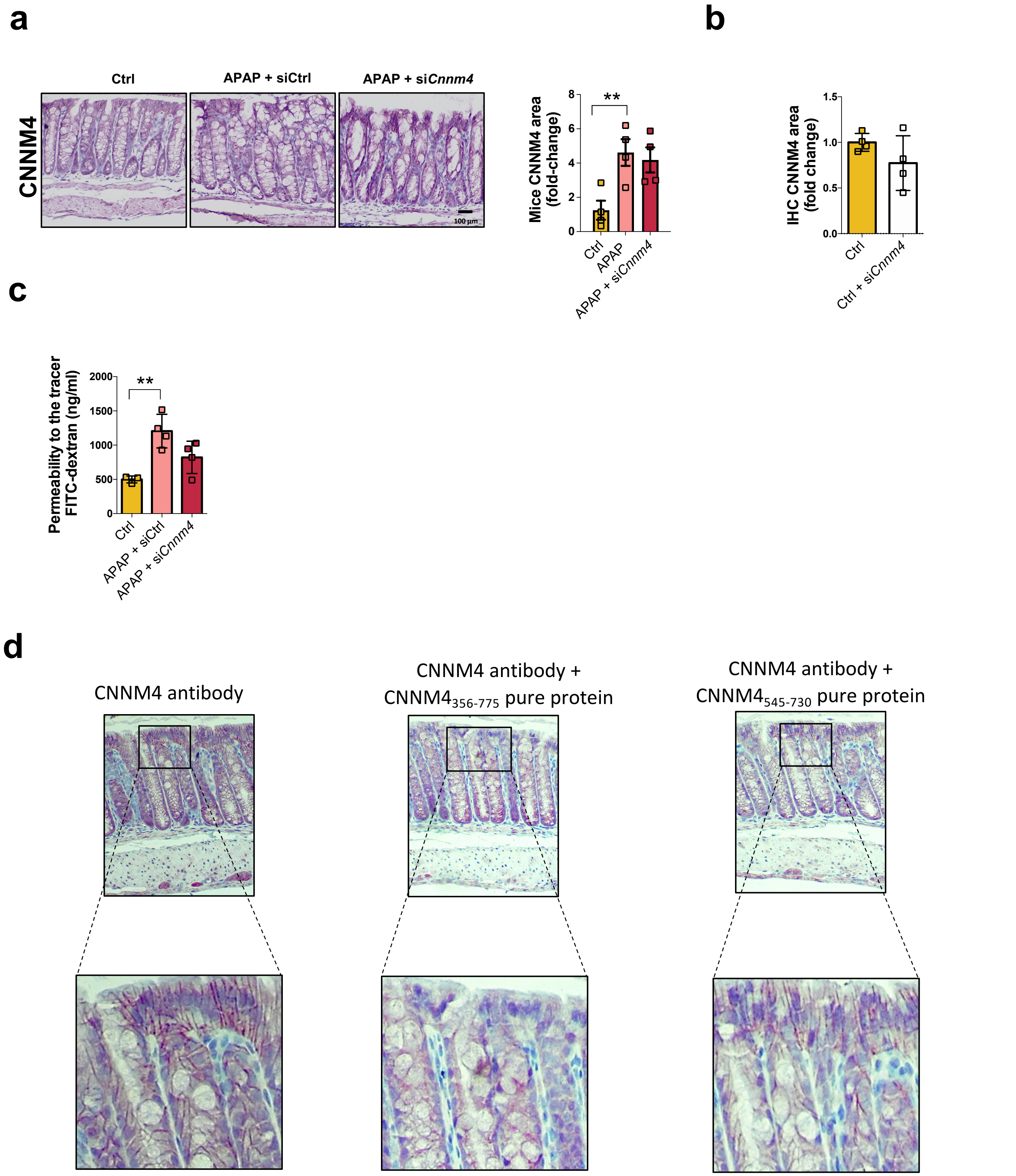
a
Ctrl
APAP + siCtrl
APAP + siCnnm4
CNNM4
100 µm
Mice CNNM4 area (fold-change)
**
Ctrl
APAP
APAP + siCnnm4
b
IHC CNNM4 area (fold change)
Ctrl
Ctrl + siCnnm4
c
Permeability to the tracer FITC-dextran (ng/ml)
**
Ctrl
APAP + siCtrl
APAP + siCnnm4
d
CNNM4 antibody
CNNM4 antibody + CNNM4356-775 pure protein
CNNM4 antibody + CNNM4545-730 pure protein

Supplemental Figure 8. Silencing *Cnnm4* specifically in the liver does not produce any negative effects on the intestinal barrier function.

A. Intestine immunohistochemical staining and respective quantification of CNNM4 was determined in WT mice under APAP overdose (n=5) compared to a healthy animal group (n=5). Scale bar correspond to 100 µm. Values are represented as mean ± SEM. ***P* < 0.01 (*P*=0.01 APAP vs Ctrl) (Student's test, two-sided). **B.** Immunohistochemical staining of the intestine and respective quantification of CNNM4 was determined in animals receiving siRNA *Cnnm4* (n=4) and compared to a control group (n=4). **C.** Dextran quantification were determined in mice serum (n=4). Values are represented as mean ± SEM. ***P* < 0.01 (*P*=0.005 APAP vs Ctrl) (Student's test, two-sided). **D.** Competitive analysis for immunohistochemistry of CNNM4 in three different conditions: IHC with 0.3 µmol/mL of CNNM4 pure protein, with only secondary antibody and with primary and secondary antibody. Source data are provided as a Source Data file.

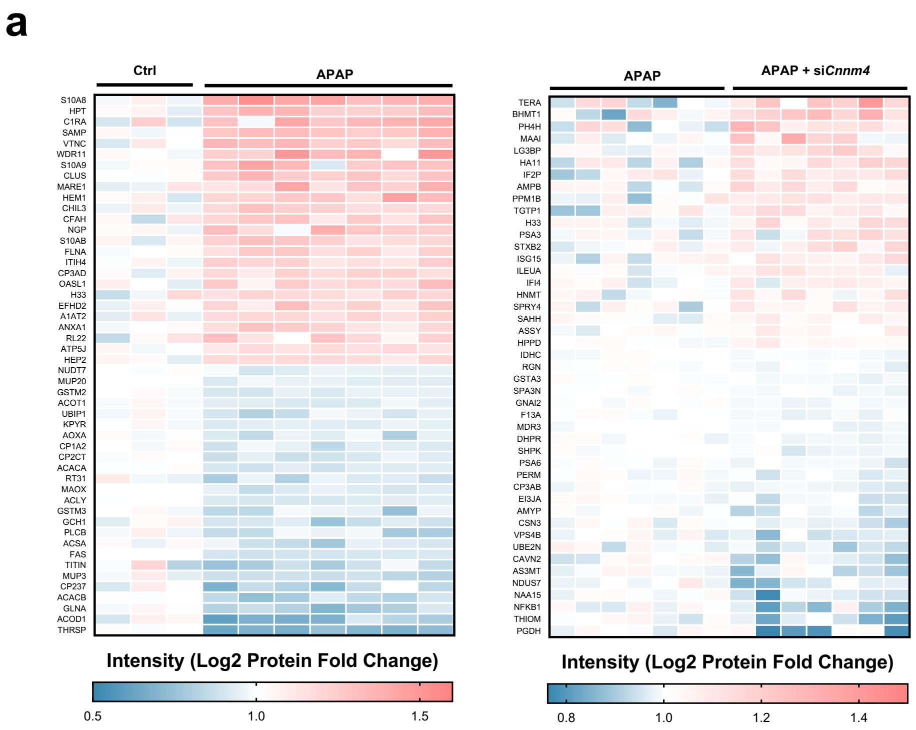
a
Ctrl
APAP
S10A8
HPT
C1RA
SAMP
VTNC
WDR11
S10A9
CLUS
MARE1
HEM1
CHIL3
CFAH
NGP
S10AB
FLNA
ITIH4
CP3AD
OASL1
H33
EFHD2
A1AT2
ANXA1
RL22
ATP5J
HEP2
NUDT7
MUP20
GSTM2
ACOT1
UBIP1
KPYR
AOXA
CP1A2
CP2CT
ACACA
RT31
MAOX
ACLY
GSTM3
GCH1
PLCB
ACSA
FAS
TITIN
MUP3
CP237
ACACB
GLNA
ACOD1
THRSP
Intensity (Log2 Protein Fold Change)
0.5
1.0
1.5
APAP
APAP + siCnnm4
TERA
BHMT1
PH4H
MAAI
LG3BP
HA11
IF2P
AMPB
PPM1B
TGTP1
H33
PSA3
STXB2
ISG15
ILEUA
IFI4
HNMT
SPRY4
SAHH
ASSY
HPPD
IDHC
RGN
GSTA3
SPA3N
GNAI2
F13A
MDR3
DHPR
SHPK
PSA6
PERM
CP3AB
EI3JA
AMYP
CSN3
VPS4B
UBE2N
CAVN2
AS3MT
NDUS7
NAA15
NFKB1
THIOM
PGDH
Intensity (Log2 Protein Fold Change)
0.8
1.0
1.2
1.4

Supplemental Figure 9. Proteomic analysis in the liver of preclinical animal models

A. Proteomics analysis by LC-MS/MS was assessed in the liver of WT mice treated with 360mg/kg APAP for 48 hours and in the last 24h mice were injected with siRNA *Cnnm4* (n=7) or an unrelated control (siCtrl) (n=7). The list of the top-50 up- and down-regulated specific proteins regulated by the absence of *Cnnm4* were analyzed. Source data are provided as a Source Data file.

SUPPLEMENTAL FIGURE 10

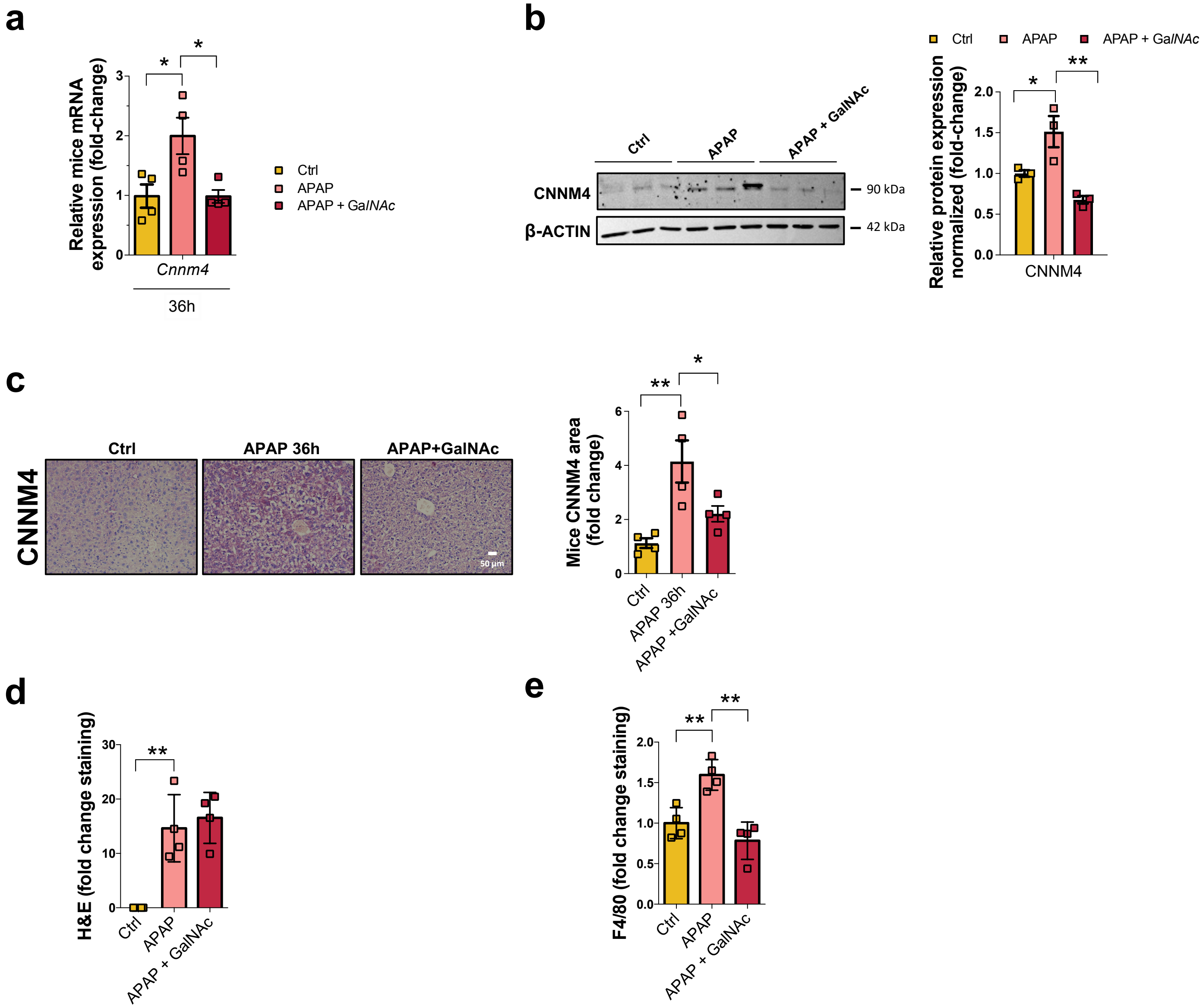

Supplemental Figure 10. Silencing *Cnnm4* prevents APAP hepatotoxicity in *in vivo* models after 36 hours of APAP overdose.

A-E. WT mice were treated with APAP 360 mg/kg by intraperitoneal injection for 36 hours (n=4) and in the last 12h 3mg/kg GalNAc *Cnnm4* siRNA (n=4) or an unrelated control (n=4) were injected subcutaneously. **A.** mRNA levels of *Cnnm4*. Values are represented as mean ± SEM. $^*P < 0.05$ (P=0.032 APAP vs Ctrl; P=0.02 APAP+GalNAc vs APAP) (Student's test, two-sided). **B.** Western blot analysis of CNNM4**,** β-ACTIN was used as a loading control. Values are represented as mean ± SEM. $^*P < 0.05$; $^{**}P < 0.01$ (P=0.059 APAP vs Ctrl; P=0.01 APAP+GalNAc vs APAP) (Student's test, two-sided). **C.** Liver immunohistochemical staining and respective quantification of CNNM4 was determined in the experimental groups of APAP 360mg/kg for 36h (n=4) and silenced with GalNac si*Cnnm4* or unrelated control (n=4) animal models compared to a healthy group (n=4). Scale bar correspond to 50 µm. Values are represented as mean ± SEM. $^*P < 0.05$; $^{**}P<0.01$ (P=0.0092 APAP vs Ctrl; P=0.05 APAP+GalNAc vs APAP) (Student's test, two-sided). **D.** Liver necrosis was assessed by H&E staining. Values are represented as mean ± SEM. $^*P<0.05$; $^{**}P<0.01$ (P=0.0032 APAP vs Ctrl) (Student's test, two-sided). **E.** Inflammation assessed by F4/80 staining in liver. Values are represented as mean ± SEM. $^{**}P<0.01$ (P=0.0046 APAP vs Ctrl; P=0.0016 APAP+GalNAc vs APAP) (Student's test, two-sided). Source data are provided as a Source Data file.

SUPPLEMENTAL FIGURE 11

Uncropped blots for Figure 4b

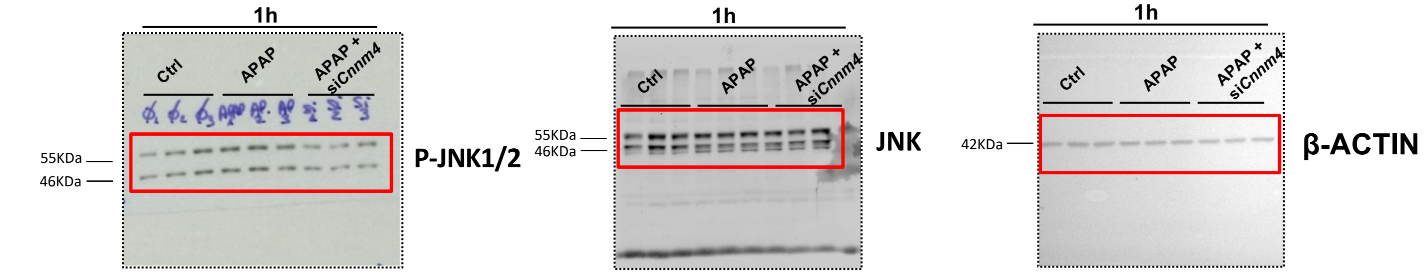

Uncropped blots for Figure 6d

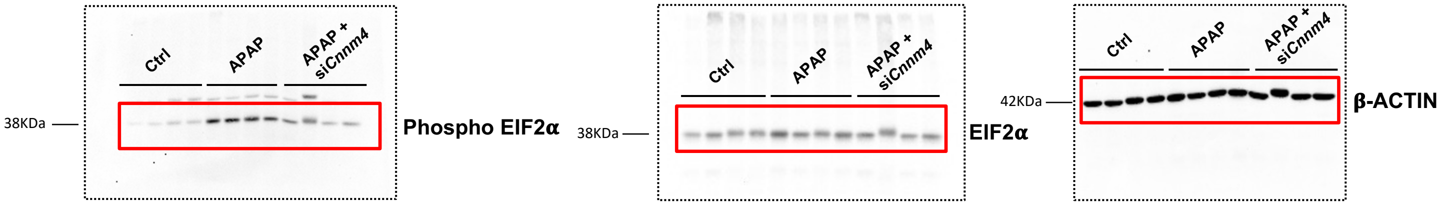

Uncropped blots for Figure 6f

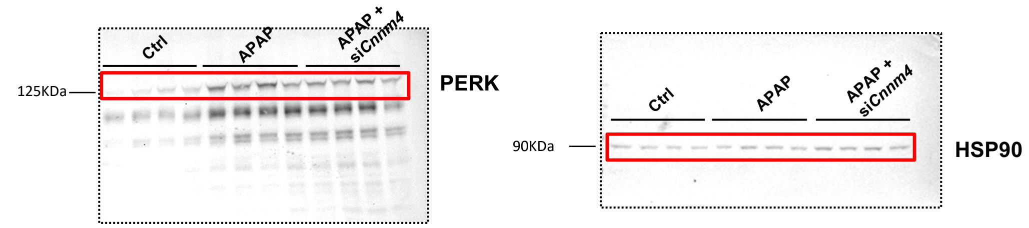

Uncropped blots for Figure 6g

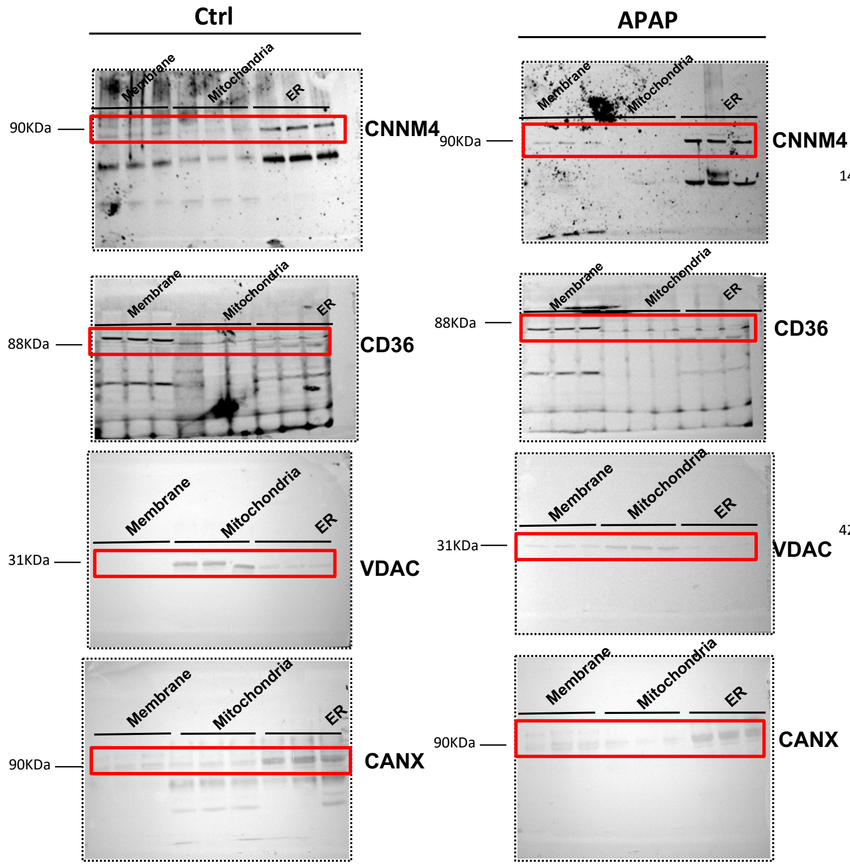

Uncropped blots for Figure 7f

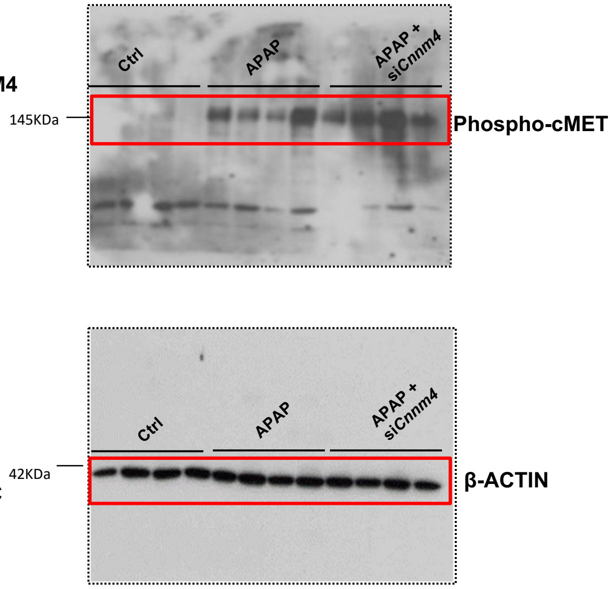

Supplemental Figure 11. Uncropped blots for figures

Uncropped blots for main figures

SUPPLEMENTAL FIGURE 12

Uncropped blots for Supp. Figure 2a

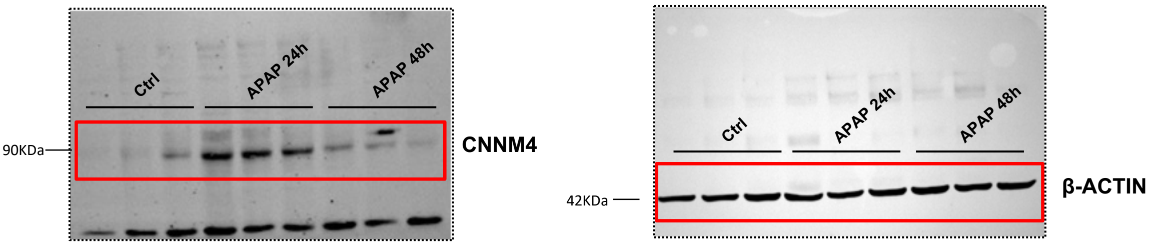

Uncropped blots for Supp. Figure 6b

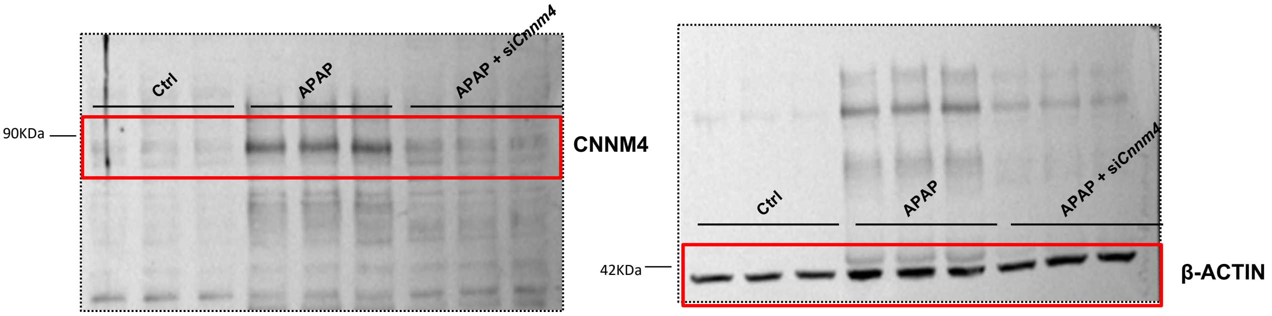

Uncropped blots for Supp. Figure 7a

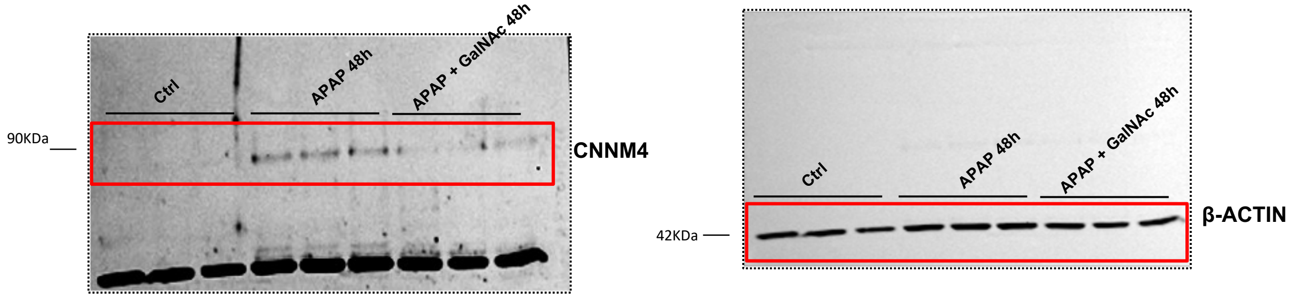

Uncropped blots for Supp. Figure 10b

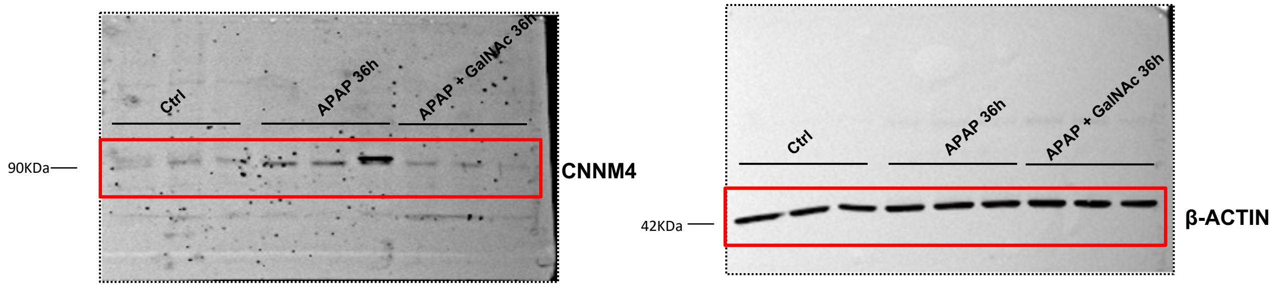

Supplemental Figure 12. Uncropped blots for Supplementary Figures

Uncropped blots for supplementary figures

Supplemental Table I. Characteristics of AILI patients

	**All patients**
n	24
Age (years)	35 ± 10.4
Gender (F/M)	13/11
Time from ingestion of APAP to hospital presentation (hours)	6.37 ± 6.2
Presentation APAP concentration (mg/L)	101 ± 65.5
Total APAP ingested (mg/kg)	261.5 ± 195.25
Serum creatinine (μmol/L)	69.75 ± 13.65
Any other drugs ingested	
Yes	19
No	5
Severity, *n* (%)	
Acute ≤ 8 hours	58.5%
Acute > 8 hours	33.25
Staggered international	17%
Supra-therapeutic	17%

Data are expressed as mean ± SD

Supplemental Table II. Characteristics of AILI patients

	**All patients**
n	13
Age (years)	36 ± 13
ALT (U/L)	2829 ± 2501
ALP (U/L)	144 ± 60
GGT (U/L)	170 ± 119
TBIL (U/L)	141 ± 83
Interval to explant (days)	3 ± 1.4
Severity, *n* (%)	
Mild	
Moderate	
Severe	13 (100%)
Fatal	

AST aspartate aminotransferase, ALT alanine aminotransferase, ALP alkaline phosphatase, GGT Gamma glutamyl transferase, TBL total bilirubin. Data are expressed as mean ± SD

Supplemental Table III. Incubation conditions, Concentration, Reference and Supplier for specific antibodies analyzed by Western Blotting.

Antibody	Supplier	Catalogue	Dilution	Incubation solution
Phospho-JNK1/JNK2 (Thr183, Tyr185)	ThermoFisher	44-682G	1:1000	TBS-Tween (0.1%)-milk (5%)
SAPK/JNK	Cell Signaling	9252S	1:1000	TBS-Tween (0.1%)-milk (5%)
Phospho EIF2α	Cell Signaling	9721S	1:1000	TBS-Tween (0.1%)-BSA (5%)
EIF2α	Cell Signaling	5324S	1:1000	TBS-Tween (0.1%)-milk (5%)
β-Actin	Sigma	A2228	1:1000	TBS-Tween (0.1%)-milk (5%)
CD36	Scbt	Sc-9154	1:1000	TBS-Tween (0.1%)-milk (5%)
VDAC	Abcam	AB15895	1:1000	TBS-Tween (0.1%)-milk (5%)
CNNM4	Abcam	AB191207	1:1000	TBS-Tween (0.1%)-milk (5%)
CNX	Scbt	Sc-23954	1:1000	TBS-Tween (0.1%)-milk (5%)
HSP90	BD Biosciences	610418	1:1000	TBS-Tween (0.1%)-milk (5%)
PERK	Cell Signaling	C33E10	1:1000	TBS-Tween (0.1%)-milk (5%)
PHOSPHO-cMET	Sigma	07-810	1:1000	TBS-Tween (0.1%)-BSA (5%)
