# Supplementary material for: Restoring cellular magnesium balance through Cyclin M4 protects against acetaminophen-induced liver damage
Source: Nat Commun. 2022 Nov 25;13:6816. doi: 10.1038/s41467-022-34262-0 (PMC9700862; doi:10.1038/s41467-022-34262-0)
Supplement: Supplementary file 3 — Description of Additional Supplementary Files [file 41467_2022_34262_MOESM3_ESM.pdf]

Description of Additional Supplementary Files

File Name: Supplementary Data 1
Description: Sequence of primers used for RT-qPCR analysis
