# Supplementary material for: Restoring cellular magnesium balance through Cyclin M4 protects against acetaminophen-induced liver damage
Source: Nat Commun. 2022 Nov 25;13:6816. doi: 10.1038/s41467-022-34262-0 (PMC9700862; doi:10.1038/s41467-022-34262-0)
Supplement: Supplementary file 5 — Reporting Summary [file 41467_2022_34262_MOESM5_ESM.pdf]

nature portfolio

Corresponding author(s): Maria Luz Martinez Chantar

Last updated by author(s): 14th June 2022

Reporting Summary

Nature Portfolio wishes to improve the reproducibility of the work that we publish. This form provides structure for consistency and transparency in reporting. For further information on Nature Portfolio policies, see our Editorial Policies and the Editorial Policy Checklist.

Statistics

For all statistical analyses, confirm that the following items are present in the figure legend, table legend, main text, or Methods section.

n/a	Confirmed	
☐	☒	The exact sample size (*n*) for each experimental group/condition, given as a discrete number and unit of measurement
☐	☒	A statement on whether measurements were taken from distinct samples or whether the same sample was measured repeatedly
☐	☒	The statistical test(s) used AND whether they are one- or two-sided<br>*Only common tests should be described solely by name; describe more complex techniques in the Methods section.*
☐	☒	A description of all covariates tested
☐	☒	A description of any assumptions or corrections, such as tests of normality and adjustment for multiple comparisons
☐	☒	A full description of the statistical parameters including central tendency (e.g. means) or other basic estimates (e.g. regression coefficient) AND variation (e.g. standard deviation) or associated estimates of uncertainty (e.g. confidence intervals)
☐	☒	For null hypothesis testing, the test statistic (e.g. *F*, *t*, *r*) with confidence intervals, effect sizes, degrees of freedom and *P* value noted<br>*Give P values as exact values whenever suitable.*
☒	☐	For Bayesian analysis, information on the choice of priors and Markov chain Monte Carlo settings
☒	☐	For hierarchical and complex designs, identification of the appropriate level for tests and full reporting of outcomes
☒	☐	Estimates of effect sizes (e.g. Cohen's *d*, Pearson's *r*), indicating how they were calculated

Our web collection on statistics for biologists contains articles on many of the points above.

Software and code

Policy information about availability of computer code

Data collection	No software was used for data collection
Data analysis	Data and statistical analysis were performed using GraphPad Prism 6 and Excel Software. Stained area percentage in immunostaining samples were calculated using FIJI (ImageJ)

For manuscripts utilizing custom algorithms or software that are central to the research but not yet described in published literature, software must be made available to editors and reviewers. We strongly encourage code deposition in a community repository (e.g. GitHub). See the Nature Portfolio guidelines for submitting code & software for further information.

Data

Policy information about availability of data

All manuscripts must include a data availability statement. This statement should provide the following information, where applicable:

- Accession codes, unique identifiers, or web links for publicly available datasets
- A description of any restrictions on data availability
- For clinical datasets or third party data, please ensure that the statement adheres to our policy

The mass spectromic data generated in this study have been deposited in the PRIDE database under accession code PXD036682.

Human research participants

Policy information about studies involving human research participants and Sex and Gender in Research.

Reporting on sex and gender	We have provided the available information about sex and gender In one cohort of patients, sex is disclosed in Supplemental Table I.
Population characteristics	The population characteristics of human research participants is included in the Supplemental Table I and II
Recruitment	The diagnosis was established by the Newcastle Hospitals NHS Foundation Trust (Newcastle, England) based on clinical data.
Ethics oversight	All trial studies in this research complies with all relevant ethical regulations. All participants in the trials of this study have signed an informed consent and they have not received a participant compensation.<br>The study EudraCT number 2017-000246-21 and ClinicalTrials.gov identifier NCT03177395 was approved by the UK medicines regulator, MHRA (25th April 2017) and West of Scotland Research Ethics Committee 1, Glasgow, UK (11th April 2017) for the twenty-four serum samples for magnesium levels determination.<br>The Newcastle Research Ethics Committee of North East Newcastle and North Tyneside approved the study with Newcastle patients tissues for the evaluation of CNNM4 expression in liver biopsies.<br>The Research Ethics Committee FIM-HEP-2016-01 approved the study including 4 serum samples of control individuals for magnesium levels determination.<br>The Research Ethics Committee of IDIVAL Cantabria 2017.052 approved the study with 3 samples from healthy individuals for immunostaining analyses.

Note that full information on the approval of the study protocol must also be provided in the manuscript.

Field-specific reporting

Please select the one below that is the best fit for your research. If you are not sure, read the appropriate sections before making your selection.

☒ Life sciences ☐ Behavioural & social sciences ☐ Ecological, evolutionary & environmental sciences

For a reference copy of the document with all sections, see nature.com/documents/nr-reporting-summary-flat.pdf

Life sciences study design

All studies must disclose on these points even when the disclosure is negative.

Sample size	DSS research sample size calculator
Data exclusions	We obtained outliers determined by using GraphPad software and Grubb's test in figures (1e,2c,6c,7a,7c,7d) and in supplemental figures (6a,8c) and were excluded for the following analysis
Replication	For cells, 4 replicates were used. For animal experiments, 4 or 5 animals were used for each experimental group. All attempts at replication were successful
Randomization	animals groups were randomly assigned
Blinding	For immunohistochemical analysis, the investigators were blinded during data collection and during analysis

Behavioural & social sciences study design

All studies must disclose on these points even when the disclosure is negative.

Study description	N/A
Research sample	N/A
Sampling strategy	N/A
Data collection	N/A
Timing	N/A

Data exclusions	N/A
Non-participation	N/A
Randomization	N/A

Ecological, evolutionary & environmental sciences study design

All studies must disclose on these points even when the disclosure is negative.

Study description	N/A
Research sample	N/A
Sampling strategy	N/A
Data collection	N/A
Timing and spatial scale	N/A
Data exclusions	N/A
Reproducibility	N/A
Randomization	N/A
Blinding	N/A

Did the study involve field work? ☐ Yes ☐ No

Field work, collection and transport

Field conditions	N/A
Location	N/A
Access & import/export	N/A
Disturbance	N/A

Reporting for specific materials, systems and methods

We require information from authors about some types of materials, experimental systems and methods used in many studies. Here, indicate whether each material, system or method listed is relevant to your study. If you are not sure if a list item applies to your research, read the appropriate section before selecting a response.

Materials & experimental systems

n/a	Involved in the study	
☐	☒	Antibodies
☐	☒	Eukaryotic cell lines
☒	☐	Palaeontology and archaeology
☐	☒	Animals and other organisms
☐	☒	Clinical data
☒	☐	Dual use research of concern

Methods

n/a	Involved in the study	
☒	☐	ChIP-seq
☒	☐	Flow cytometry
☒	☐	MRI-based neuroimaging

Antibodies

Antibodies used	Phospho-JNK1/JNK2 (Thr183,Tyr 185) (ThermoFisher, 44-682G) 1:1000 dilution; SAPK/JNK (Cell Signaling, 9252S) 1:1000 dilution;

Antibodies used	Phospho EIF? (Cell Signaling, 9721S) 1:1000 Dilution; Eif2? (Cell Signaling, 5324S) 1:1000 dilution; ?-Actin (Sigma Aldrich, A2228); CD36 (Sbct, Sc-9154) 1:1000 dilution; VDAC (Abcam, AB15895) 1:1000 dilution; CNNM4 (Abcam, AB191297) 1:1000 dilution, CNX (Sbct, Sc-23954) 1:1000 dilution; HSP90 (BD Biosciences, 610418) 1:1000 dilution; PERK (Cell Signaling, C33E10) 1:1000 dilution; PHOSPHO-cMET (Sigma Aldrich, 07-810) 1:1000 dilution.<br>The secondary antibodies were anti-mouse (Cell Signaling, 7076S) and anti-rabbit (Cell Signaling, 7074S) used in a dilution 1:5000.
Validation	Antibodies were validated for the suppliers which are Indicated in Supplemental Table III

Eukaryotic cell lines

Policy information about cell lines and Sex and Gender in Research

Cell line source(s)	Primary hepatocytes from mice and the human liver epithelial cells (Thle2) from ATCC? (CRL-2706)
Authentication	Thle-2 cell line was not authenticated
Mycoplasma contamination	The cell line was not tested for mycoplasma contamination.
Commonly misidentified lines (See ICLAC register)	no

Palaeontology and Archaeology

Specimen provenance	N/A
Specimen deposition	N/A
Dating methods	N/A

☐ Tick this box to confirm that the raw and calibrated dates are available in the paper or in Supplementary Information.

Ethics oversight	N/A

Note that full information on the approval of the study protocol must also be provided in the manuscript.

Animals and other research organisms

Policy information about studies involving animals; ARRIVE guidelines recommended for reporting animal research, and Sex and Gender in Research

Laboratory animals	Male adult (three-month old) C57BL/6 mice were acquired from Charles River (St Germain sur l'Arbresle, France) and maintained at CIC bioGUNE animal facility. Animal procedures were approved by the CIC bioGUNE Institutional Animal Care and Use Committee and the local authority (Diputación de Bizkaia).<br>Mice were kept in a temperature-controlled animal facility (AAALAC-accredited) with 12-hour light/dark cycles, and fed a standard diet (Harlan Tekland) with water and libitum.
Wild animals	No wild animals were used in the study.
Reporting on sex	male
Field-collected samples	No field collected samples were used in the study, since information given here is not relevant to this field.
Ethics oversight	All animal experimentation was carried out in accordance with Spanish Guide for the Care and use of Laboratory animals, and with International Animal Care and Use of Committee. Animal procedures were approved by the CIC bioGUNE Institutional Animal Care and Use Committee and the local authority (Diputación de Bizkaia).

Note that full information on the approval of the study protocol must also be provided in the manuscript.

Clinical data

Policy information about clinical studies

All manuscripts should comply with the ICMJE guidelines for publication of clinical research and a completed CONSORT checklist must be included with all submissions.

Clinical trial registration	NCT03177395
Study protocol	EudraCT number 2017-000246-21
Data collection	N/A

Outcomes	N/A

Dual use research of concern

Policy information about dual use research of concern

Hazards

Could the accidental, deliberate or reckless misuse of agents or technologies generated in the work, or the application of information presented in the manuscript, pose a threat to:

No	Yes	
☒	☐	Public health
☒	☐	National security
☒	☐	Crops and/or livestock
☒	☐	Ecosystems
☒	☐	Any other significant area

Experiments of concern

Does the work involve any of these experiments of concern:

No	Yes	
☒	☐	Demonstrate how to render a vaccine ineffective
☒	☐	Confer resistance to therapeutically useful antibiotics or antiviral agents
☒	☐	Enhance the virulence of a pathogen or render a nonpathogen virulent
☒	☐	Increase transmissibility of a pathogen
☒	☐	Alter the host range of a pathogen
☒	☐	Enable evasion of diagnostic/detection modalities
☒	☐	Enable the weaponization of a biological agent or toxin
☒	☐	Any other potentially harmful combination of experiments and agents

ChIP-seq

Data deposition

☐ Confirm that both raw and final processed data have been deposited in a public database such as GEO.

☐ Confirm that you have deposited or provided access to graph files (e.g. BED files) for the called peaks.

Data access links<br>*May remain private before publication.*	N/A
Files in database submission	N/A
Genome browser session<br>(e.g. UCSC)	N/A

Methodology

Replicates	N/A
Sequencing depth	N/A
Antibodies	N/A
Peak calling parameters	N/A
Data quality	N/A
Software	N/A

Flow Cytometry

Plots

Confirm that:

- [ ] The axis labels state the marker and fluorochrome used (e.g. CD4-FITC).
- [ ] The axis scales are clearly visible. Include numbers along axes only for bottom left plot of group (a 'group' is an analysis of identical markers).
- [ ] All plots are contour plots with outliers or pseudocolor plots.
- [ ] A numerical value for number of cells or percentage (with statistics) is provided.

Methodology

Sample preparation	N/A
Instrument	N/A
Software	N/A
Cell population abundance	N/A
Gating strategy	N/A

- [ ] Tick this box to confirm that a figure exemplifying the gating strategy is provided in the Supplementary Information.

Magnetic resonance imaging

Experimental design

Design type	N/A
Design specifications	N/A
Behavioral performance measures	N/A

Acquisition

Imaging type(s)	N/A
Field strength	N/A
Sequence & imaging parameters	N/A
Area of acquisition	N/A

Diffusion MRI ☐ Used ☐ Not used

Preprocessing

Preprocessing software	N/A
Normalization	N/A
Normalization template	N/A
Noise and artifact removal	N/A
Volume censoring	N/A

Statistical modeling & inference

Model type and settings	N/A
Effect(s) tested	N/A

Specify type of analysis: ☐ Whole brain ☐ ROI-based ☐ Both

Statistic type for inference (See Eklund et al. 2016)	N/A
Correction	N/A

Models & analysis

n/a	Involved in the study
☐	☐ Functional and/or effective connectivity
☐	☐ Graph analysis
☐	☐ Multivariate modeling or predictive analysis

Functional and/or effective connectivity	N/A
Graph analysis	N/A
Multivariate modeling and predictive analysis	N/A
